# Simultaneous genotyping for human platelet antigen systems and *HLA-A* and *HLA-B* loci by targeted next-generation sequencing

**DOI:** 10.3389/fimmu.2022.945994

**Published:** 2022-09-29

**Authors:** Jielin Wang, Xuan You, Yanmin He, Xiaozhen Hong, Ji He, Sudan Tao, Faming Zhu

**Affiliations:** ^1^ Human Leukocyte Antigen Typing Laboratory, Blood Center of Zhejiang Province, Hangzhou, China; ^2^ Human Leukocyte Antigen Typing Laboratory, Key Laboratory of Blood Safety Research, Hangzhou, China

**Keywords:** human leukocyte antigen, human platelet antigens, platelet transfusion refractoriness, next-generation sequencing, platelet blood donors

## Abstract

In order to treat the alloimmunization platelet transfusion refractoriness (PTR), human leukocyte antigen (HLA)-type and/or human platelet antigen (HPA)-type matched platelets between donors and patients are usually used. Therefore, genotyping of *HLA-A* and *HLA-B* loci, as well as HPA systems, for donors and patients, is of great significance. However, there is a rare report of genotyping for *HLA-A* and *HLA-B* loci as well as HPA systems at the same time. In this study, a high-throughput method for simultaneous genotyping of *HLA-A* and *HLA-B* loci, as well as HPA genotyping, was developed. A RNA capture probe panel was designed covering all exon sequences of the *GP1BA*, *GP1BB*, *ITGA2*, *CD109*, *ITGB3*, and *ITGA2B* genes and *HLA-A* and *HLA-B* loci. The *HLA-A*, *HLA-B*, and 34 HPA systems were genotyped using a targeted next-generation sequencing (NGS) method. The genotypes of the *HLA-A* and *HLA-B* loci, as well as the HPA, were assigned based on the nucleotides in the polymorphism sites. Using the NGS method, 204 unrelated blood specimens were successfully genotyped for all 34 HPA systems as well as *HLA-A* and *HLA-B* loci. The accuracy of the NGS method was 100%. Only HPA-2, HPA-3, HPA-5, HPA-6w, HPA-15, and HPA-21w showed polymorphism with frequencies of 0.9412, 0.6863, 0.9853, 0.9779, 0.4314, and 0.9951 for a allele, respectively. Thirty-two single nucleotide variants (SNVs) were detected. Of them, 12 SNVs can lead to amino acid change. HLA-A*11:01 and HLA-B*46:01 are the most common alleles for *HLA-A* and *HLA-B* loci. A targeted next-generation sequencing method for simultaneously genotyping HPA systems and *HLA-A* and *HLA-B* loci was first established, which could be used to create a database of HLA-typed and/or HPA-typed unrelated donors.

## Introduction

There are a variety of alloantigen expression on the human platelet membrane surface, including some erythrocyte blood group antigens, human leukocyte antigen (HLA) class I proteins, human platelet antigens (HPA), and CD36 antigen (1–4). Nonetheless, the blood group system antigens, HLA class I antigen, and CD36 antigen in the human platelet membrane are shared with other cells (5–7). Classical HLA class I genes consist of *HLA-A*, *HLA-B*, and *HLA-C* loci. It has been reported that the *HLA-A*, *HLA-B*, and *HLA-C* loci are highly polymorphic, and their distribution in various populations is different (8). To date, 35 HPA have been identified and officially nominated by the International Platelet Immunology Nomenclature Committee of the International Society of Blood Transfusion (ISBT) (9). All HPA are located on the GPIIb/IIIa, GPIb/V/IX, GPIa/IIa, and CD109 platelet membrane glycoprotein complexes (1, 10). The antigens of the HPA systems showed a certain degree of polymorphism, but HPA a and b antigens were only found in HPA-1 to HPA-6w, HPA-15, and HPA-21w in the Chinese Han population (11).

Due to the difference in platelets’ alloantigen expression in the individuals, it can trigger a corresponding immune response to form antibodies through transfusion, pregnancy pathways, and allotransplantation. Antibodies against *HLA-A* and *HLA-B*, HPA, and CD36 antigens are responsible for some clinical syndromes and transfusion-related conditions, such as platelet transfusion refractoriness (PTR), posttransfusion purpura (PTP), and neonatal alloimmune thrombocytopenia (NAIT) (1, 12, 13). Currently, the transfusion of platelets for both prophylaxis and treatment of bleeding is relevant to all areas of clinical medicine. PTR is a common complication of patients receiving multiple transfusions, which is defined as an unsatisfactory posttransfusion platelet count increment. PTR can be separated into immune and nonimmune causes (14). In the alloimmunization PTR, most cases can be found with anti-HLA, anti-HPA, and/or anti-CD36 (15–17). In order to treat and prevent the alloimmunization of PTR, a common method was used using HLA-typed and/or HPA-typed matched platelets (18, 19). However, this needs to establish a database for HLA-typed and/or HPA-typed unrelated donors. It shows that 5,000, 18,000, and 25,000 donor candidates would be necessary to find at least five completely compatible donors in the Japanese, European Caucasoid, and North American Caucasoid populations, respectively (19). Although platelets’ membrane surfaces express the *HLA-A*, *HLA-B*, and *HLA-C* molecules, platelet donors are not routinely matched for HLA-C antigen during the HLA match platelet procedure (20).

In order to genotype for HLA-typed and/or HPA-typed unrelated donors, it needs to provide the corresponding methods for *HLA-A* and *HLA-B* loci, and *HPA* systems. Currently, many methods have been developed to detect *HLA-A* and *HLA-B* loci and *HPA* systems, including PCR sequence-specific primers (PCR-SSP), PCR sequence-based typing (PCR-SBT), and real-time PCR (11, 21–23). However, these methods always detect single gene separately and need too many wells and multiple amplification for the test. Now, next-generation sequencing (NGS) has been widely used for genotyping *HLA* loci. When compared with PCR-SBT, it has some advantages in terms of throughput and cost (24–26). Besides short-read sequencing, long-read sequencing technologies have been reported, such as single-molecule real-time sequencing (SMRT; Pacific Biosciences (PacBio)) and nanopore sequencing (Oxford Nanopore Technologies (ONT)) (27–29). Compared with short-read sequencing, these two long-read technologies provide lower per-read accuracy and require high data processing equipment. Furthermore, some studies have reported that the NGS method was used for HPA genotyping (30–34). Vorholt et al. reported an amplicon-based approach to genotyping for HPA-1 to HPA-5, HPA-9w, HPA-10w, HPA-16w, HPA-19w, HPA-27w, and HPA-34w, which required being amplified into 12 different fragments in each specimen (33). Davey et al. developed a targeted enrichment, high-sensitivity HaloPlex assay for 29 HPA systems, and 47 samples were sequenced (34). However, there is a rare report of simultaneously genotyping *HLA-A* and *HLA-B* loci as well as HPA systems. Here, in order to establish a database for HLA-typed and/or HPA-typed unrelated donors, a simultaneous genotyping method for *HLA-A* and *HLA-B loci* and HPA-1–HPA-30 and HPA-32–HPA-35 systems was established based on the target enrichment technology. The frequencies of genotypes and alleles of 34 HPA systems and *HLA-A* and *HLA-B* loci were also analyzed in the Chinese platelet blood donors.

## Materials and methods

### Study specimens

In total, 204 unrelated blood specimens were collected from healthy platelet blood donors who have at least donated the platelet three times at the Blood Center of Zhejiang Province, China. The ethnic background of all individuals is Zhejiang Han. Informed consent was obtained from all participants. This study was approved by the Ethical Scientific Committee of the Blood Center of Zhejiang Province, China (2020-005). From each platelet donor, 5 ml peripheral blood with EDTA anticoagulant was collected. To validate the accuracy of the method, nine HPA reference specimens from the 14th, 16th, and 20th Platelet Immunology Workshop of ISBT have been chosen for detection, which contained HPA-1ab, HPA-1bb, and HPA-4ab genotypes, respectively.

### Genomic DNA extraction

The genomic DNA was extracted using a commercial DNA extraction reagent kit (RBC Bioscience, Taiwan) and an automatic Magcore nucleic acid extraction instrument (RBC Bioscience, Taiwan) according to the manufacturer’s instructions. The OD260/280 ratio of the DNA was 1.6 to 1.8. The final DNA concentration was adjusted to 30 ng/μl.

### The probe panel design

A RNA capture probe panel covering all exon sequences of the *GP1BA*, *GP1BB*, *ITGA2*, *CD109*, *ITGB3*, and *ITGA2B* genes and *HLA-A* and *HLA-B* loci was designed based on the GRCh37 reference sequence and synthesized by a commercial company (Lianchuan Biotechnology Co. Ltd., Hangzhou, China). This panel included 498 nonoverlapping probes, covering 97.88% of target regions. The length of each probe was 120 bp, and all probes were designed by end-to-end tiling to match the reference sequence. The 5′ end of all capture probes is coupled with biotin and then all probes were mixed. The information on the covered region in the gene for HPA and *HLA-A* and *HLA-B* genotyping are listed in **Table 1**. The sequences of all probes are listed in **Supplementary Table S1**.

**Table 1:** The information on covered region in the gene for HPA and *HLA-A* and *HLA-B* genotyping.

Gene	Protein	Exons number	Chromosome location (GRChr37:hg19)
*CD109*	CD109	33	chr6:74405808-74538041
*GP1BA*	GPIbA	2	chr17:4835570-4838325
*GP1BB*	GPIbB	2	chr22:19711066-19712297
*ITGA2*	GPIa	30	chr5:52285156-52390609
*ITGA2B*	GPIIb	30	chr17:42449549-42466969
*ITGB3*	GPIIIa	15	chr17:45331208-45390077
*HLA-A*	HLA-A	8	chr6:29910247-29913661
*HLA-B*	HLA-B	7	chr6:2655402-2658781

chr, chromosome.

### Genomic DNA fragmentation and amplification

DNA fragmentation and amplification were prepared from genomic DNA using the VariantBaits™ Target Enrichment System (Lianchuan Biotechnology Co., Ltd., Hangzhou, China) according to the manufacturer’s instructions. Briefly, a total of 200 ng genomic DNA for each specimen was sheared to 200–250 bp by a focused ultrasonicator (M220-Covaris, Auburn, MA, USA) at 4°C, followed by end-repairing, A-tailing, index ligation, and purification. The purification DNA was then amplified in a 50 µl reaction system, including 20 µl DNA fragments, 5 µl primers, and 25 µl DNA polymerase master mixture, by initial denaturation at 98°C for 45 s, followed by 7 cycles at 98°C for 15 s, 60°C for 30 s, 72°C for 30 s, and final extension at 72°C for 1 min. The PCR amplicons with 200–500 bp length were obtained by size selection using purification magnetic beads (ThermoFisher, San Jose, CA, USA). The PCR amplicon concentration and quality were determined by the Qubit instrument (ThermoFisher, San Jose, CA, USA) and the Agilent 4200 Bioanalyzer (Agilent Technologies Inc., Santa Clara, CA, USA).

### Hybridization in solution and target-capture

Every four indexed PCR amplicons (500 ng) were pooled into one tube for hybridization and subsequently added 7.5 µl of different blocking solutions. The tube was evaporated in a vacuum freeze drier (Heto-Holten, ThermoFisher Scientific, San Jose, CA, USA) at 40°C. In total, 7.5 μl 2× hybridization buffer and 3 μl blocking solution B were added to the dry tube and incubated at 95°C for 5 min and 65°C for 5 min. After, 4.5 µl VariantBaits™ biotinylated probes (Lianchuan Biotechnology Co. Ltd., Hangzhou, China) were added, and the hybridization was performed at 65°C for 16 to 24 h. In this step, PCR amplicons that contained the target sequences of the HPA systems and *HLA-A* and *HLA-B* loci would be specifically hybridized into biotinylated capture probes. In the next reaction, 40 µl of Dynabeads™ MyOne™ Streptavidin T1 magnetic beads (ThermoFisher, San Jose, CA, USA) was used to separate and purify the captured product from the above hybridization system, and the product was resuspended using 20 µl nuclease-free water. The 20 µl separated DNA was amplified with a 50 µl reaction mix containing 5 µl primer mixture and 25 µl DNA polymerase master mixture, performed as follows: 98°C for 45 s; 16 cycles of 98°C for 15 s, 60°C for 30 s, and 72°C for 30 s; and a final extension at 72°C for 1 min. The PCR products were purified with magnetic beads, and the quality was detected by the Agilent 4200 Bioanalyzer.

### Sequencing

The aforementioned PCR products from different tubes were pooled into one new tube equally, which formed the DNA libraries. The quality and size of the pooled library were detected by the Agilent Bioanalyzer 4200. The concentration of the library was determined by the Qubit instrument, prepared to a final concentration of 12 pmol/L, and then sequenced using a MiSeq instrument (Illumina, San Diego, CA, USA) with a standard v2 Reagent Kit (2*318 cycle; Illumina, San Diego, CA, USA).

### Data analysis

The FASTQ files generated by the MiSeq instrument were analyzed for all exon sequences, including the nucleotides in the polymorphism sites of the HPA systems using CLC benchwork 23.11 (Qiagen Company, Stockach, Germany) according to the manufacturer’s instructions. The sequences of the *GP1BA* (NG_008767), *GP1BB* (NG_007974), *ITGA2* (NG_008330), *CD109* (NG_0033971), *ITGB3* (NG_008332), and *ITGA2B* (NG_008331) were set as the reference sequences, respectively. The FASTQ files were aligned with the different reference sequences using CLC benchwork 23.11. Sequence data with quality over Q30 and depth of coverage over 30× were accepted. The genotypes for HPA-1 to HPA-35 (except for HPA-31) were assigned manually according to the nucleotides in the polymorphism sites from the versiti-HPA database (www.versiti.org). The genotypes of *HLA-A* and *HLA-B* loci were assigned using the TypeStream Visual Software version 2.0 (One Lambda Inc., Canoga Park, CA, USA).

### Genotyping HPA-1–HPA-35 systems by PCR-SBT

In order to validate the results of the established NGS method, the specimens were also genotyped for HPA-1–HPA-28 systems using a PCR-SBT method according to our previous report (11). Three new primer pairs were added for genotyping HPA-29 to HPA-35 systems, and the procedure is the same as in our previous report except for the primers. In brief, 21 of the specific primers were divided into seven groups; therefore, one group has three primer pairs and mixed into one well. The nucleotide sequence of each HPA system was amplified using the primer mixture, and the amplicons were then Sanger sequenced using a Big Dye Terminator v3.1 cycle sequencing kit (ThermoFisher Scientific, Shanghai, China). The genotypes for HPA systems were assigned according to the nucleotides in the polymorphism sites of the HPA systems.

### Genotyping *HLA-A* and *HLA-B* loci using a commercial NGS method

The *HLA-A* and *HLA-B* loci were also genotyped using an AllType NGS 9-Loci Amplification Kit (One Lambda Inc.) according to the manufacturer’s instructions. The *HLA-A* and *HLA-B* genotypes were assigned using the TypeStream Visual Software version 2.0 with the *IMGT/HLA* Database version 3.46.0.0 (One Lambda Inc.) as previously reported (35).

### Statistical analysis

A Hardy–Weinberg equilibrium (HWE) was determined for each HPA system using the Chi-square test. *p*-values of less than 0.05 were considered statistically significant.

## Results

### Validate the results of the established NGS method

A total of 204 specimens from the blood donors were detected by the NGS method. The results of the *HLA-A* and *HLA-B* loci and HPAs in all specimens consisted of those of the commercial NGS or PCR-SBT in-house. The accuracy of the NGS method for HLA and HPA genotyping was 100%. The HPA genotypes in the nine reference specimens by the NGS method were in concordance with the reference results and demonstrated in **Supplementary Table S2**.

Some quality parameters of the NGS procedure were as below. All the observed sequence lengths were 150 bps, and the distribution of GC-content fits normal distribution, and the relative GC-content of a sequence of R1 and R2 in 20%–80% were 99.81% and 99.54%, respectively. The relative N-content of a sequence less than 1% of R1 and R2 were 99.95% and 99.99%. In addition, the distribution of average sequence quality scores over 30 of R1 and R2 was 98.78% and 98.08%, respectively. The mean value of the reading depth in the exon regions for each gene is shown in **Supplementary Table S3**. Meanwhile, the mean reading depth frequency value of minor alleles of single nucleotide variants (SNVs) in the heterozygous positions is shown in **Supplementary Table S4**.

### The distribution of 34 HPA systems by the NGS method

The genotype distributions of the 34 HPA systems (HPA-31w was not analyzed by the NGS method) were fitted with Hardy–Weinberg equilibrium (*p* > 0.05). Among these HPA systems, only HPA-2, HPA-3, HPA-5, HPA-6bw, HPA-15bw, and HPA-21bw systems observed polymorphism in this study. However, a/a homozygote individuals were found in the other 28 HPA systems. The genotypes and allele frequencies of the systems with polymorphism are listed in **Table 2**. Among these 34 HPA systems, HPA-3 and HPA-15 systems showed high polymorphisms, with frequencies of 0.6863 and 0.3137 for HPA-3a and HPA-3b alleles and 0.4314 and 0.5686 for HPA-15a and HPA-15b alleles, respectively.

**Table 2:** The HPA genotypes and allele frequencies in the Chinese platelet blood donors (*n* = 204).

HPA system	Genotype number			Allele frequency		*X_2_*	*p*
	aa	ab	bb	a^a^	b^a^		
HPA-2	182	20	2	0.9412	0.0588	2.6784	>0.05
HPA-3	99	82	23	0.6863	0.3137	0.9026	>0.05
HPA-5	198	6	0	0.9853	0.0147	0.0454	>0.05
HPA-6w	195	9	0	0.9779	0.0221	0.1038	>0.05
HPA-15	46	84	74	0.4314	0.5686	5.2655	>0.05
HPA-21w	202	2	0	0.9951	0.0049	0.0050	>0.05

**^a^** a and b are the alleles of the HPA system.

### Variants detected in the *ITGB3*, *ITGA2B*, *ITGA2*, *CD109*, and *GP1BA* genes

Thirty-two SNVs were detected in the exon regions of the *ITGB3*, *ITGA2B*, *ITGA2*, *CD109*, and *GP1BA* genes. Only one SNV has not existed in the dbSNP database. No SNV was found in the *GP1BB* gene. The position of these SNVs and allele frequencies are shown in **Table 3**. Of these 32 SNVs identified, 12 SNVs can result in an amino acid change, while the other SNVs are synonymous changes. A new SNV site, c.2878G>A located on the exon 23 of *CD109*, can result in an amino acid change, p.Gly960Ser, the novel SNV has been submitted to Genbank, the Accession number is OP434394. The amino acid was located in the A-macroglobulin receptor-binding domain according to the Simple Modular Architecture Research Tool (SMART) analysis based on the CD109 antigen (Q6YHK3-4). The probably damaging effect was predicted for this SNV using the PolyPhen-2 software (v2.2.3r406) *in silico*, and the score was 1.000 (score 0.000–0.452 = benign, 0.453–0.956 = possibly damaging, 0.957–1.000 = probably damaging).

**Table 3:** The SNVs in the exon regions of the *ITGB3*, *ITGA2B*, *ITGA2*, *CD109*, and *GP1BA* genes.

Gene	Location	Nucleotide change	MAF	Amino acid substitution	Predicted domains, repeats, motifs, and features	SNP ID	MAF of East Asian
*ITGB3*	Exon 6	882T>C	0.292	–	VWA domain	rs5919	0.196
	Exon 8	1143A>C	0.380	–	VWA domain	rs15908	0.490
	Exon 10	1533A>G	0.177	–		rs4642	0.399
	Exon 10	1545G>A	0.306	–		rs4634	0.405
	Exon 10	1641C>T	0.005	–		rs185135224	0.007
	Exon 11	1902C>T	0.009	–		rs149823724	0.014
*ITGA2B*	Exon 29	3063C>T	0.319	–	Transmembrane region	rs5910	0.423
*ITGA2*	Exon 3	327G>A	0.015	Met109Ile	Integrin alpha region	rs188816090	0.010
	Exon 7	759C>T	0.108	–	VWA domain	rs1126643	0.296
	Exon 7	789T>C	0.017	–	VWA domain	rs1139484	0.020
	Exon 7	825G>A	0.265	–	VWA domain	rs1062535	0.297
	Exon 8	993A>G	0.020	–		rs3212523	0.019
	Exon 21	2780A>G	0.007	Asn927Ser		rs2287870	0.025
	Exon 26	3252C>T	0.286	–	Transmembrane region	rs2303122	0.352
	Exon 26	3324T>C	0.005	–	Transmembrane region	rs772014693	0.010
*CD109*	Exon 4	645C>T	0.228	–		rs6453696	0.132
	Exon 17	1923G>T	0.007	Leu641Phe		rs7742662	0.008
	Exon 21	2390A>G	0.355	Asn797Ser		rs2351528	0.409
	Exon 21	2533G>A	0.348	Val845Ile		rs5023688	0.388
	Exon 23	2878G>A	0.005	Gly960Ser	A2M_receptor-binding domain	NA	
	Exon 29	3722C>T	0.404	Thr1241Met		rs2917862	0.476
	Exon 31	3945T>C	0.995	–		rs772548630	0
	Exon 32	4173G>T	0.042	–	A2M_receptor-binding domain	rs2917887	0.041
*GP1BA*	Exon 2	1074A>G	0.159	–		rs6067	0.198
	Exon 2	1282T>C	0.255	Ser428Pro		rs187469311	0.200
	Exon 2	1291G>A	0.096	Ala431Thr		rs1379346895	0
	Exon 2	1293C>T	0.056	–		rs1172798146	0
	Exon 2	1296C>A	0.0833	–		rs553749201	0.070
	Exon 2	1305C>T	0.074	–		rs761456793	0.060
	Exon 2	1321T>C	0.135	Ser441Pro		rs12947958	0
	Exon 2	1326G>T	0.107	Glu442Asp		rs1159453070	0.001
	Exon 2	1344C>T	0.145	–		rs41466145	0.230

The frequency for the East Asian population is cited from the dbSNP database.

SNVs, single nucleotide variants; NA, not found in the dbSNP database; MAF, minor allele frequency.

### The distribution for *HLA-A* and *HLA-B* loci, respectively

The numbers of *HLA-A* and *HLA-B* alleles were 23 and 49 in the 204 specimens, respectively. The HLA-A*11:01 allele and HLA-B*46:01 allele are the most common alleles for each locus, with a frequency of 22.55% and 14.22%, respectively. The allele distributions of the *HLA-A* and *HLA-B* loci are listed in **Supplementary Table S5**.

## Discussion

Thrombocytopenic patients with immune-based PTR would have significantly increased the risk of a major spontaneous or life-threatening bleed. In order to treat these patients, one of the common methods is to provide cross-match-compatible or HLA-matched/compatible platelet units (15, 18). In order to provide matching platelets, it needs a large number of donors with known HLA and/or HPA genotypes (18, 19). At present, many DNA-based methods have been used for HLA and/or HPA genotyping in platelet donors (20–28, 30, 31). It has been reported that PCR-SBT and NGS methods have been routinely used for HLA genotyping in the laboratory (24–26, 35, 36). The NGS methods for HLA genotyping are divided into targeted and amplicon-based methods (34, 35). Classical *HLA-A*, *HLA-B*, and *HLA-C* molecules were all expressed in the platelet, but the HLA-C molecule was expressed at a low level. Therefore, only *HLA-A* and *HLA-B* loci were routinely analyzed to search for HLA-matched/compatible platelet units (20). The HPA genotyping methods included PCR-SSP, TaqMan assay, PCR-SBT, and NGS (22, 23, 33, 34). However, there is a rare study for HPA and HLA genotyping simultaneously. Now genotyping for all HPA systems and *HLA-A* and *HLA-B* loci needs multiple PCR amplifications and/or sequencing reactions using the PCR-SSP and PCR-SBT methods, which do not simultaneously detected the all HPA systems and *HLA-A* and *HLA-B* loci. Here, we report an NGS method for simultaneously genotyping HPA-1~35 systems (except HPA-31w) and *HLA-A* and *HLA-B* loci in platelet donors.

In this study, target-enrichment panel probes for the *HLA-A, HLA-B, ITGB3, ITGA2B, ITGA2, CD109, GP1BA, and GP1BB* genes were designed based on GRCh37 as a reference. Due to the HPA-31w polymorphism, the site is located in the *GP9* gene and it was not nominated when we initiated this study (10). Therefore, the target probes were not for the *GP9* gene, and the protocol cannot assign the HPA-31w system. The design probes overlapped one by one to cover all exon regions. Therefore, all known human platelet antigens except for HPA-31w and novel variations in the encoding regions can be detected. In the panel, the probes are RNA-based, which has better hybridization efficiency and stability to DNA complexes compared to DNA probes (37, 38). However, the RNA probes are expensive and require a lower temperature to store. In the hybridization reaction, four tubes were pooled into one to assure the volume was less than 31 μl after pooling; otherwise, it will affect hybridization efficiency. In order to validate the results of the established NGS method, the specimens were genotyped for all HPAs by a PCR-SBT method in-house and for *HLA-A* and *HLA-B* loci using a commercial NGS method. It showed that the accuracy of the established NGS method for HLA and HPA was 100%.

The accuracy of the NGS method depends on some influence factors. Davey et al. reported the results of the NGS method for HPA were in concordance 100% with historical HPA genotypes (34). However, Vorholt et al. reported that the concordance with TaqMan assay and NGS results for HPAs was 99.8%, and the discrepancies between them were due to allele dropout in real-time PCR (33). It is generally considered that a depth of coverage of over 30× is necessary for some SNV detection (39). Due to the depth of coverage being different among the genes in most NGS methods, it needs to assure a depth of coverage of over 30× for each gene. The accuracy can be improved with increased coverage (40). However, the coverage can also be influenced by the GC content, which may affect the efficiency of PCR. There was also evidence that the same volume had more coverage than the same mole (33), but the specimens that were mixed in the same mole were better than the same volume in our experiment. In addition, we found a repetitive sequence in exon 2 of the *GP1BA* gene, which can affect the analysis accuracy when using the CLC benchwork 23.11. Therefore, the SNVs in the *GP1BA* gene by the NGS method were also confirmed by PCR-SBT in-house. Besides the depth of coverage, the breadth of coverage also helps to ensure that sensitivity and specificity are sufficient for supporting variant detection. However, the coverage profiles may not be uniform and a lack of coverage in key regions, such as exons, may affect the accuracy of the typing. Although software analysis programs will usually have built-in filters to define the minimum coverage required for accurate typing, certain circumstances may permit going below this threshold, such as when the polymorphisms of two alleles of a locus are phased or when the region with low coverage does not affect the genotyping (i.e., introns, untranslated regions). Furthermore, besides the depth of coverage, for HLA typing data analysis, the assessment of adequate allele balance is important for detecting issues due to allele dropout.

The distribution of *HLA-A*, *HLA-B*, and HPA systems was similar to those of our previous reports (11, 35). Furthermore, some SNVs were detected in the coding region, which is located in the structural domain of the platelet membrane glycoproteins (**Table 3**). c.882T>C and c.1143A>C in the *ITGB3* were both located in the VWA domain (41), which are synonymous changes. c.3063C>T in the *ITGA2B* is located in the transmembrane region (42), a hydrophobic region of the protein. c.327G>A in the *ITAG2* is located in the integrin alpha region of the GPIa (43); c.759C>T, c.789T>C, c.825G>A, and c.993A>G in the *ITAG2* are located in the VWA domain of the GPIa; and c.3252C>T and c.3324T>C are located in the transmembrane region of the GPIa. c.4173G>T in the *CD109* is located in the A2M_receptor-binding domain, which is the receptor-binding domain (RBD) of alpha-2-macroglobulin proteins (44, 45). Therefore, some SNVs may affect the domain function, but this needs further study.

In summary, a targeted NGS method can be successfully established to genotype simultaneously 34 HPA systems as well as *HLA-A* and *HLA-B* loci. Its advantages include high throughput and simultaneous testing for *HLA-A*, *HLA-B*, and HPA genotypes. The method can be used to establish a database bank of HLA-typed and/or HPA-typed unrelated donors, which can help provide matching platelets for PTR, PTP, and NAIT patients.

## Data availability statement

The datasets presented in this study can be found in online repositories. The names of the repository/repositories and accession number(s) can be found in the article/**Supplementary Material**.

## Ethics statement

Written informed consent was obtained from the individual(s) for the publication of any potentially identifiable images or data included in this article.

## Author contributions

JW, XY and YH performed the experiments, collected the data, and wrote the rough draft. XH and JH analyzed the data. ST and FZ interpreted the results and drafted the paper. All authors contributed to the article and approved the submitted version.

## Funding

This work was supported by the Science Research Foundation of Zhejiang Healthy Bureau (2021KY137 and WKJ-ZJ-2021).

## Conflict of interest

The authors declare that the research was conducted in the absence of any commercial or financial relationships that could be construed as a potential conflict of interest.

## Publisher’s note

All claims expressed in this article are solely those of the authors and do not necessarily represent those of their affiliated organizations, or those of the publisher, the editors and the reviewers. Any product that may be evaluated in this article, or claim that may be made by its manufacturer, is not guaranteed or endorsed by the publisher.

## Abbreviations

HPA, human platelet antigen; PCR-SBT, polymerase chain reaction sequence-based typing; PTR, platelet transfusion refractoriness; PTP, posttransfusion purpura; NAIT, neonatal alloimmune thrombocytopenia; NGS, next-generation sequencing.
