# Supplementary material for: Simultaneous genotyping for human platelet antigen systems and HLA-A and HLA-B loci by targeted next-generation sequencing
Source: Front Immunol. 2022 Sep 29;13:945994. doi: 10.3389/fimmu.2022.945994 (PMC9575554; doi:10.3389/fimmu.2022.945994)
Supplement: Supplementary file 2 [file Table_2.docx]

**Supplemental table 2** The genotype of nine specimens with known HPAs by NGS.

| HPA | Specimen ID number | | | | | | | | |
| --- | --- | --- | --- | --- | --- | --- | --- | --- | --- |
|  | 1 | 2 | 3 | 4 | 5 | 6 | 7 | 8 | 9 |
| HPA-1 | ab | bb | bb | bb | aa | aa | aa | aa | ab |
| HPA-2 | ab | aa | aa | aa | aa | aa | aa | aa | aa |
| HPA-3 | aa | ab | aa | ab | bb | aa | aa | ab | bb |
| HPA-4 | aa | aa | aa | aa | ab | bb | aa | aa | aa |
| HPA-5 | ab | aa | aa | bb | ab | aa | ab | bb | ab |
| HPA-6w | aa | aa | aa | aa | aa | aa | aa | aa | aa |
| HPA-7 w | aa | aa | aa | aa | aa | aa | aa | aa | aa |
| HPA-8 w | aa | aa | aa | aa | aa | aa | aa | aa | aa |
| HPA-9 w | aa | aa | aa | aa | aa | aa | aa | aa | ab |
| HPA-10 w | aa | aa | aa | aa | aa | aa | aa | aa | aa |
| HPA-11 w | aa | aa | aa | aa | aa | aa | aa | aa | aa |
| HPA-12 w | aa | aa | aa | aa | aa | aa | aa | aa | aa |
| HPA-13 w | aa | aa | aa | aa | aa | aa | aa | aa | aa |
| HPA-14 w | aa | aa | aa | aa | aa | aa | aa | aa | aa |
| HPA-15 | ab | ab | ab | aa | bb | aa | ab | bb | bb |
| HPA-16 w | aa | aa | aa | aa | aa | aa | aa | aa | aa |
| HPA-17 w | aa | aa | aa | aa | aa | aa | aa | aa | aa |
| HPA-18 w | aa | aa | aa | aa | aa | aa | aa | aa | aa |
| HPA-19 w | aa | aa | aa | aa | aa | aa | aa | aa | aa |
| HPA-20 w | aa | aa | aa | aa | aa | aa | aa | aa | aa |
| HPA-21 w | aa | aa | aa | aa | aa | aa | aa | aa | aa |
| HPA-22 w | aa | aa | aa | aa | aa | aa | aa | aa | aa |
| HPA-23 w | aa | aa | aa | aa | aa | aa | aa | aa | aa |
| HPA-24 w | aa | aa | aa | aa | aa | aa | aa | aa | aa |
| HPA-25 w | aa | aa | aa | aa | aa | aa | aa | aa | aa |
| HPA-26 w | aa | aa | aa | aa | aa | aa | aa | aa | aa |
| HPA-27 w | aa | aa | aa | aa | aa | aa | aa | aa | aa |
| HPA-28 w | aa | aa | aa | aa | aa | aa | aa | aa | aa |
| HPA-29 w | aa | aa | aa | aa | aa | aa | aa | aa | aa |
| HPA-30 w | aa | aa | aa | aa | aa | aa | aa | aa | aa |
| HPA-32 w | aa | aa | aa | aa | aa | aa | aa | aa | aa |
| HPA-33 w | aa | aa | aa | aa | aa | aa | aa | aa | aa |
| HPA-35 w | aa | aa | aa | aa | aa | aa | aa | aa | aa |

**Supplement table 3** The mean value of the minor reading depth in the exon regions for each gene

|  | *ITGB3* | *ITGA2* | *ITGA2B* | *CD109* | *GP1BB* | *GP1BA* | *HLA-A* | *HLA-B* |
| --- | --- | --- | --- | --- | --- | --- | --- | --- |
| exon 1 | 124 | 631 | 987 | 544 | 169 | 657 | 139 | 454 |
| exon 2 | 494 | 336 | 929 | 1032 | 389 | 1311 | 222 | 194 |
| exon 3 | 794 | 452 | 686 | 216 | - | - | 95 | 233 |
| exon 4 | 1023 | 712 | 109 | 506 | - | - | 313 | 44 |
| exon 5 | 972 | 419 | 448 | 307 | - | - | 106 | 367 |
| exon 6 | 753 | 295 | 1199 | 267 | - | - | 82 | 270 |
| exon 7 | 260 | 487 | 1078 | 281 | - | - | 64 | 323 |
| exon 8 | 707 | 550 | 739 | 657 | - | - | - | - |
| exon 9 | 411 | 473 | 642 | 429 | - | - | - | - |
| exon 10 | 977 | 486 | 569 | 208 | - | - | - | - |
| exon 11 | 887 | 698 | 1204 | 762 | - | - | - | - |
| exon 12 | 684 | 369 | 947 | 645 | - | - | - | - |
| exon 13 | 285 | 481 | 648 | 932 | - | - | - | - |
| exon 14 | 574 | 720 | 534 | 512 | - | - | - | - |
| exon 15 | 1775 | 686 | 830 | 344 | - | - | - | - |
| exon 16 | - | 217 | 762 | 398 | - | - | - | - |
| exon 17 | - | 564 | 711 | 523 | - | - | - | - |
| exon 18 | - | 694 | 675 | 358 | - | - | - | - |
| exon 19 | - | 445 | 713 | 341 | - | - | - | - |
| exon 20 | - | 308 | 857 | 208 | - | - | - | - |
| exon 21 | - | 758 | 1198 | 1223 | - | - | - | - |
| exon 22 | - | 682 | 730 | 316 | - | - | - | - |
| exon 23 | - | 801 | 761 | 428 | - | - | - | - |
| exon 24 | - | 272 | 1118 | 424 | - | - | - | - |
| exon 25 | - | 313 | 1428 | 568 | - | - | - | - |
| exon 26 | - | 310 | 537 | 661 | - | - | - | - |
| exon 27 | - | 326 | 694 | 631 | - | - | - | - |
| exon 28 | - | 420 | 877 | 596 | - | - | - | - |
| exon 29 | - | 282 | 765 | 340 | - | - | - | - |
| exon 30 | - | 1131 | 1508 | 199 | - | - | - | - |
| exon 31 | - | - | - | 709 | - | - | - | - |
| exon 32 | - | - | - | 331 | - | - | - | - |
| exon 33 | - | - | - | 1237 | - | - | - | - |

**Supplement table 4** The mean value of the percentage of the minor allele in the heterozygous positions

| Gene | Location | Nucleotide change | mean value of the percentage of the minor allele in the heterozygous positions（%） |
| --- | --- | --- | --- |
| *ITGB3* | exon 6 | 882T>C | 45.23 |
|  | exon 8 | 1143A>C | 47.46 |
|  | exon 10 | 1533A>G | 47.28 |
|  | exon 10 | 1545G>A | 46.89 |
|  | exon 10 | 1641C>T | 39.31 |
|  | exon 11 | 1902C>T | 44.42 |
| *ITGA2B* | exon 29 | 3063C>T | 49.16 |
| *ITGA2* | exon 3 | 327G>A | 40.84 |
|  | exon 7 | 759C>T | 44.44 |
|  | exon 7 | 789T>C | 51.09 |
|  | exon 7 | 825G>A | 43.91 |
|  | exon 8 | 993A>G | 42.96 |
|  | exon 21 | 2780A>G | 52.22 |
|  | exon 26 | 3252C>T | 46.61 |
|  | exon 26 | 3324T>C | 54.09 |
| *CD109* | exon 4 | 645C>T | 45.66 |
|  | exon 17 | 1923G>T | 52.08 |
|  | exon 21 | 2390A>G | 49.88 |
|  | exon 21 | 2533G>A | 39.60 |
|  | exon 23 | 2878G>A | 47.57 |
|  | exon 29 | 3722C>T | 42.95 |
|  | exon 31 | 3945T>C | 54.13 |
|  | exon 32 | 4173G>T | 55.78 |
| *GP1BA* | exon 2 | 1074A>G | 41.90 |
|  | exon 2 | 1282T>C | 46.36 |
|  | exon 2 | 1291G>A | 40.35 |
|  | exon 2 | 1293C>T | 36.84 |
|  | exon 2 | 1296C>A | 45.69 |
|  | exon 2 | 1305C>T | 53.94 |
|  | exon 2 | 1321T>C | 32.52 |
|  | exon 2 | 1326G>T | 53.94 |
|  | exon 2 | 1344C>T | 41.82 |

**Supplemental table 5** The number and frequency of the *HLA-A* and *-B* alleles

| ***HLA-A*** | **Number** | **Frequency (%)** | ***HLA-B*** | **Number** | **Frequency (%)** |
| --- | --- | --- | --- | --- | --- |
| *01:01* | 21 | 5.15 | *07:02* | 10 | 2.45 |
| *02:01* | 41 | 10.05 | *07:05* | 2 | 0.49 |
| *02:03* | 12 | 2.94 | *08:01* | 2 | 0.49 |
| *02:05* | 3 | 0.74 | *13:01* | 21 | 5.15 |
| *02:06* | 23 | 5.64 | *13:02* | 15 | 3.68 |
| *02:07* | 36 | 8.82 | *15:01* | 17 | 4.17 |
| *02:10* | 3 | 0.74 | *15:02* | 17 | 4.17 |
| *02:99* | 1 | 0.25 | *15:11* | 8 | 1.96 |
| *03:01* | 7 | 1.72 | *15:18* | 5 | 1.23 |
| *11:01* | 92 | 22.55 | *15:25* | 1 | 0.25 |
| *11:02* | 7 | 1.72 | *15:27* | 4 | 0.98 |
| *23:01* | 1 | 0.25 | *15:58* | 1 | 0.25 |
| *24:02* | 71 | 17.40 | *27:04* | 1 | 0.25 |
| *24:04* | 1 | 0.25 | *27:05* | 2 | 0.49 |
| *26:01* | 12 | 2.94 | *27:07* | 1 | 0.25 |
| *29:01* | 1 | 0.25 | *35:01* | 9 | 2.21 |
| *29:02* | 1 | 0.25 | *35:02* | 1 | 0.25 |
| *30:01* | 14 | 3.43 | *35:03* | 2 | 0.49 |
| *30:02* | 1 | 0.25 | *37:01* | 9 | 2.21 |
| *31:01* | 14 | 3.43 | *38:01* | 1 | 0.25 |
| *32:01* | 3 | 0.74 | *38:02* | 8 | 1.96 |
| *33:03* | 41 | 10.05 | *39:01* | 10 | 2.45 |
| *68:01* | 2 | 0.49 | *40:01* | 41 | 10.05 |
|  |  |  | *40:02* | 6 | 1.47 |
|  |  |  | *40:03* | 2 | 0.49 |
|  |  |  | *40:06* | 17 | 4.17 |
|  |  |  | *41:01* | 1 | 0.25 |
|  |  |  | *44:03* | 6 | 1.47 |
|  |  |  | *44:05* | 1 | 0.25 |
|  |  |  | *45:01* | 1 | 0.25 |
|  |  |  | *46:01* | 61 | 14.95 |
|  |  |  | *47:01* | 1 | 0.25 |
|  |  |  | *48:01* | 9 | 2.21 |
|  |  |  | *50:01* | 3 | 0.74 |
|  |  |  | *51:01* | 21 | 5.15 |
|  |  |  | *51:02* | 7 | 1.72 |
|  |  |  | *52:01* | 7 | 1.72 |
|  |  |  | *53:01* | 1 | 0.25 |
|  |  |  | *54:01* | 18 | 4.41 |
|  |  |  | *55:01* | 1 | 0.25 |
|  |  |  | *55:02* | 13 | 3.19 |
|  |  |  | *55:04* | 1 | 0.25 |
|  |  |  | *55:12* | 2 | 0.49 |
|  |  |  | *56:01* | 1 | 0.25 |
|  |  |  | *56:03* | 1 | 0.25 |
|  |  |  | *57:01* | 5 | 1.23 |
|  |  |  | *58:01* | 29 | 7.11 |
|  |  |  | *67:01* | 5 | 1.23 |
